# Inhibition of Phosphatidylinositol 3-kinease suppresses formation and progression of experimental abdominal aortic aneurysms

**DOI:** 10.1038/s41598-017-15207-w

**Published:** 2017-11-09

**Authors:** Jing Yu, Rui Liu, Jianhua Huang, lixin Wang, Wei Wang

**Affiliations:** 1 Department of Vascular Surgery, Xiangya Hospital, Central South University, Changsha, Hunan China; 2 Department of Vascular Surgery, Zhongshan Hospital, Fudan University, Shanghai, China

## Abstract

Accumulating evidence suggests an important role of Phosphatidylinositol 3-kinease (PI3K) pathway in inflammatory cells infiltration. Given  the essential role of inflammatory cells infiltration during the formation and progression of abdominal aortic aneurysm (AAA), to investigate the possibility of preventing AAA formation and progression via targeting PI3K is anticipated. Here, experimental AAAs was created in rats by transient intraluminal porcine pancreatic elastase (PPE) infusion into the infrarenal aorta firstly. AAAs rats were administrated with vehicle or Wortmannin during the period of day 0 to day 28 after PPE infusion. The aortic diameter of rats treated with Wortmannin was significantly smaller than those treated with vehicle. Meanwhile, Elastin destruction score and SMC destruction score were significantly decreased in rats treated with Wortmannin. Furthermore, histological analysis revealed infiltration of inflammatory cells were significantly reduced in rats treated with Wortmannin. Finally, the mRNA expression of PI3K and protein expression of pAKT in human abdominal aneurismal aorta tissues was elevated as compare to normal aorta. Our study revealed that PI3K inhibitor suppresses experimental AAAs formation and progression, through mechanisms likely related to impairing inflammation cells infiltration and median elastin degradation. These findings indicated that PI3K inhibitor may hold substantial translation value for AAA diseases.

## Introduction

Abdominal aortic aneurysm (AAA) is a common degenerative disease of the abdominal aorta that leads to its dilatation and to rupture. The mortality of ruptured AAA approximates 90% if it is untreated and around 40% if surgery is performed^1^. Many randomized trial evidences show that the aneurysm diameter is the main determinant for rupture, and surgical repair is considered appropriate when the aortic diameter exceeds 55 mm^2^. However, to date, no pharmacology strategy has been proven effective in limiting aneurysm progression or reducing risk of rupture when the aortic diameter is less than 55 mm^3,4^. Although the pathogenesis for aneurysm formation is poorly understood, human AAAs is histologically accompanied by chronic inflammation of the aorta wall. Inflammatory cell infiltration and angiogenesis are essential for the abnormal dilatation of the abdominal aorta^5^.

Phosphatidylinositol-3-kinase (PI3K) plays a vital role in the cancer cell apoptosis and tumor suppression of some human cancer^6^. PI3K/AKT signaling pathway has diverse biological actions, and is involved in cell survival, apoptosis, growth, energy metabolism and migration^7–10^. Documents revealed an important role of PI3K in inflammatory cells infiltration^11,12^, which suggested a potential relationship between PI3K function and initiation and progression of AAAs^13^. Thus, to explore the function of PI3K in initiation and progression of AAA is anticipated.

To address this question, a traditional experimental AAA model in Sprague-Dawley rats was build following intra-aortic infusion of porcine pancreatic elastase (PPE)^14,15^. Wortmannin, a PI3K pathway inhibitor (Selleckchem, China) was employed to block the PI3K/AKT pathway in experimental AAAs. Our present study indicated the possibility that limiting AAA formation and progression via targeting PI3K pathway, which highlight the potential value of PI3K inhibitor for clinical AAA disease management.

## Result

### PI3K and pAKT are up-regulated in experimental aneurismal aorta

**Figure 1:**
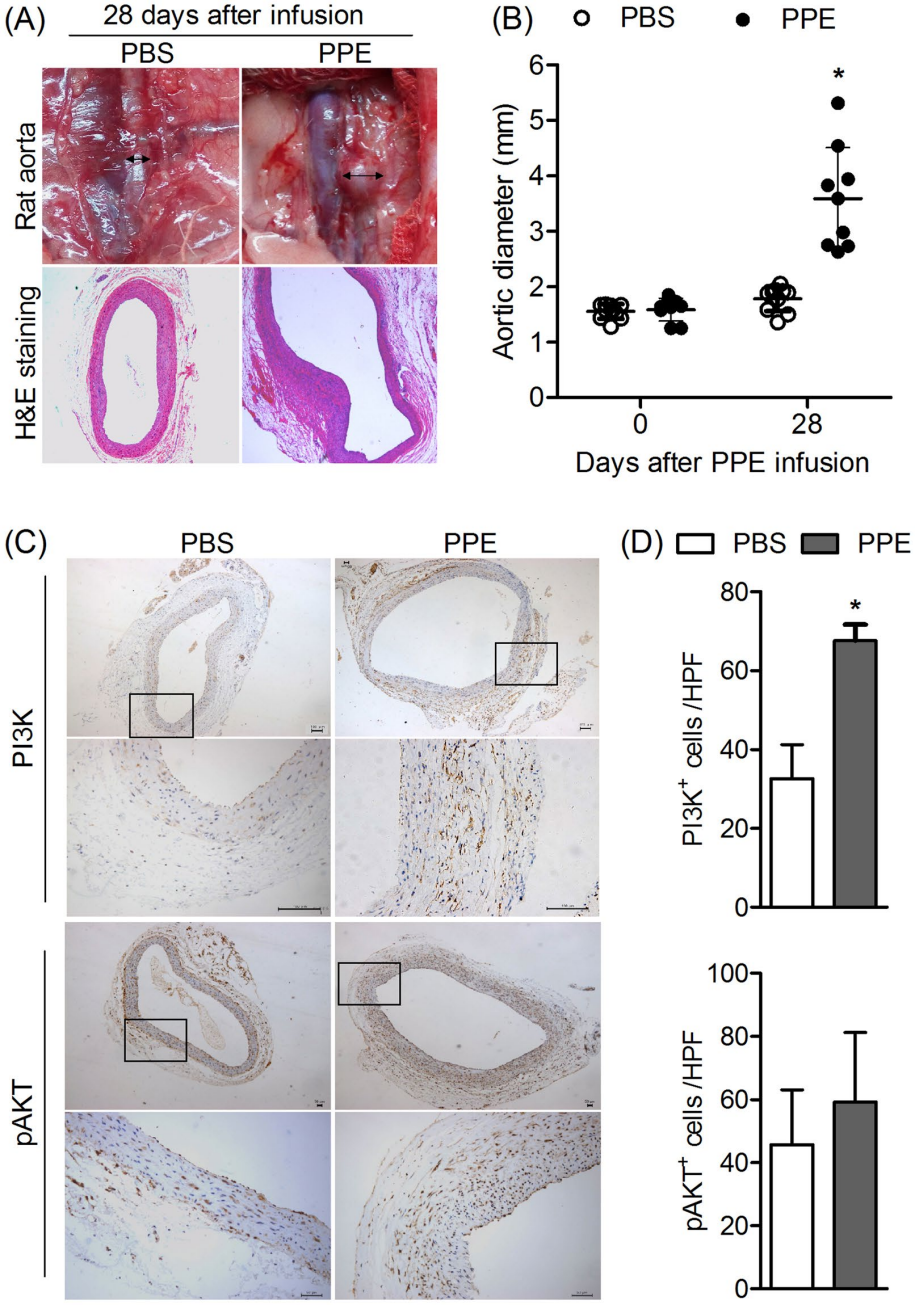
Construction of PPE infusion experimental AAA in rats and the expression of PI3K and pAKT in aneurismal aorta. 8–12 week-old male Sprague-Dawley rats were employed for PPE-induced AAA model. Vernier caliper was employed to measure the diameter of aorta directly underwent laparotomy at day 0 and day 28 after PPE infusion. An AAA was defined as a 50% increase in aortic diameter compared with baseline. (**A**) Representative images of aorta and H&E staining from PPE-infused rats and PBS-infusion rats at day 28. (**B**) Aortic diameter at day 0 and day 28 after PPE infusion (n = 9) or PBS infusion (n = 10) was measured, Data show mean ± SD and two way ANOVA test, *P < 0.05. (**C**) Aortic sections from rats 28 days after PPE or PBS infusion were collected and immunoassayed with an antibody against PI3K or pAKT, the representative aortic histology images for PI3K and pAKT staining was shown in LPF (50×) and HPF (200×) respectively. (**D**) Numbers of positive immunoassayed cells were compared between PPE infusion rats and PBS infusion rats. Data present as mean and SD per HPF. n = 9–10 in each group. Nonparametric Mann-Whitney test. *P < 0.05 vs PBS group. LPF, Low-power field; HPF, High-power field.

To investigate the potential role of PI3K in progression of AAA, an experimental abdominal aortic aneurysm was built up in rats firstly as described in previous^15^. The rat aorta all developed aortic aneurysm in PPE group whereas few changes in PBS group (Fig.  1A,B). IHC was employed to analyze the expression of PI3K and pAKT. As shown in Fig.  1C,D, the PI3K and pAKT in rat aorta were elevated in PPE group as compare to PBS group respectively (*P* < 0.05).

### PI3K inhibitor treatment prevents the formation and progression of AAA

**Figure 2:**
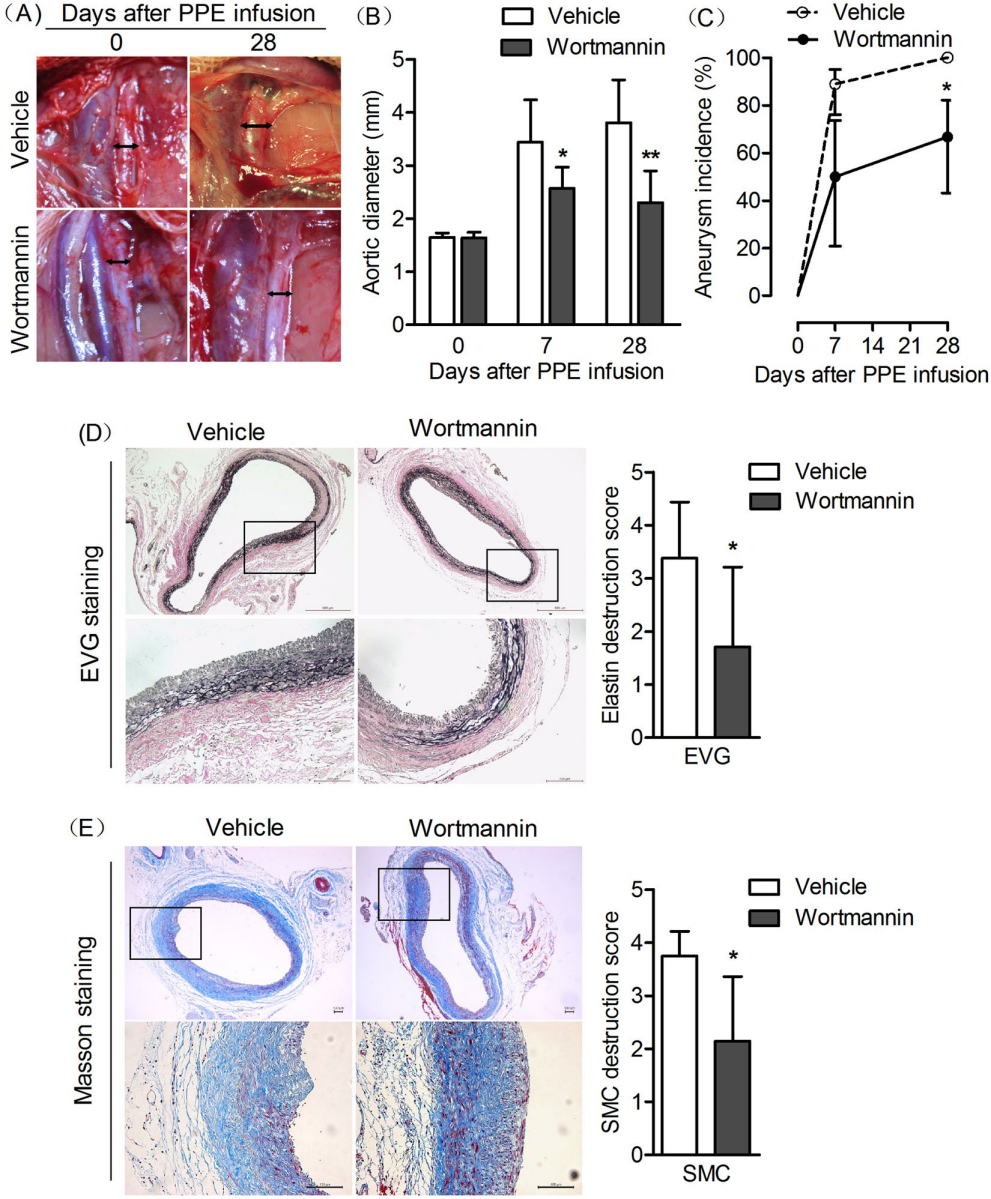
PI3K inhibitor treatment inhibits AAA formation and progression. Male SD rats were administrated with intraperitoneal injection of Wortmannin at a dose of 0.5 mg/kg/day (dissolved in DMSO) from day 0 to day 28 after PPE infusion. AAAs diameter for each rat was measured directly underwent laparotomy. (**A**) Representative images of aorta from Wortmannin treatment or Vehicle treatment in rats at day 0 and day 28. (**B**) Aortic diameters in rats after PPE infusion treated with Wortmannin (n = 12) or Vehicle (n = 9), ANOVA followed by Newman-Keuls post-test, *P < 0.05 and ** *P* < 0.01 vs vehicle group. (**C**) The incidence of AAA in PPE-infused rats with Wortmannin or Vehicle treatment was evaluated. Kaplan-Meier analysis. *P < 0.05 vs vehicle group. (**D**,**E**) Histological staining of EVG or Masson was performed to evaluate the development of AAAs. Representative aortic histology images for elastin and SMCs in each group. Medial elastin fragmentation and SMCs destruction were scored as mild 1 to severe 5 using a histology grading system. Data are presented with Mean and SD, n = 9–12 in each group. Nonparametric Mann-Whitney test. * *P* < 0.05 vs vehicle group.

Based on the increased expression of PI3K and pAKT in aneurismal aorta, we questioned whether systemic inhibition of PI3K could prevent the AAA formation and progression. To address our question, rats was administered by intraperitoneal injection with vehicle or PI3K inhibitor (Wortmannin at dose of 0.4 mg/ml/day dissolved in DMSO) during the period of day 0 to day 28 after PPE infusion. AAAs diameter for each rat was measured directly by using open operation. PPE infusion rats treated with Wortmannin produced significantly smaller aneurysms as compare to PPE infusion rats treated with vehicle. In time points of aneurysm progression (7 and 28 day), Mean aortic diameters after PPE infusion in Wortmannin treatment group was significantly smaller than PPE infusion in vehicle treatment group (*P* < 0.05). AAAs developed in eight rats (89%, 8/9) within 7 days and in all rats (100%, 9/9) with 28 days in vehicle-treated groups, whereas AAA developed in six rats (50%, 6/12) within 7 days and eight rats (67%, 8/12) within 28 days treated with Wortmannin respectively (*P* < 0.05). Furthermore, EVG and Masson staining on aortic sections were performed. As showed in Fig.  2D,E, both elastin destruction score and SMC destruction score were significantly decreased in rats treated with Wortmannin (*P* < 0.05). Together, these results indicated that PI3K inhibitor treatment inhibits experimental AAA formation and progression.

### PI3K inhibitor treatment attenuates the inflammatory cell infiltration in AAAs

**Figure 3:**
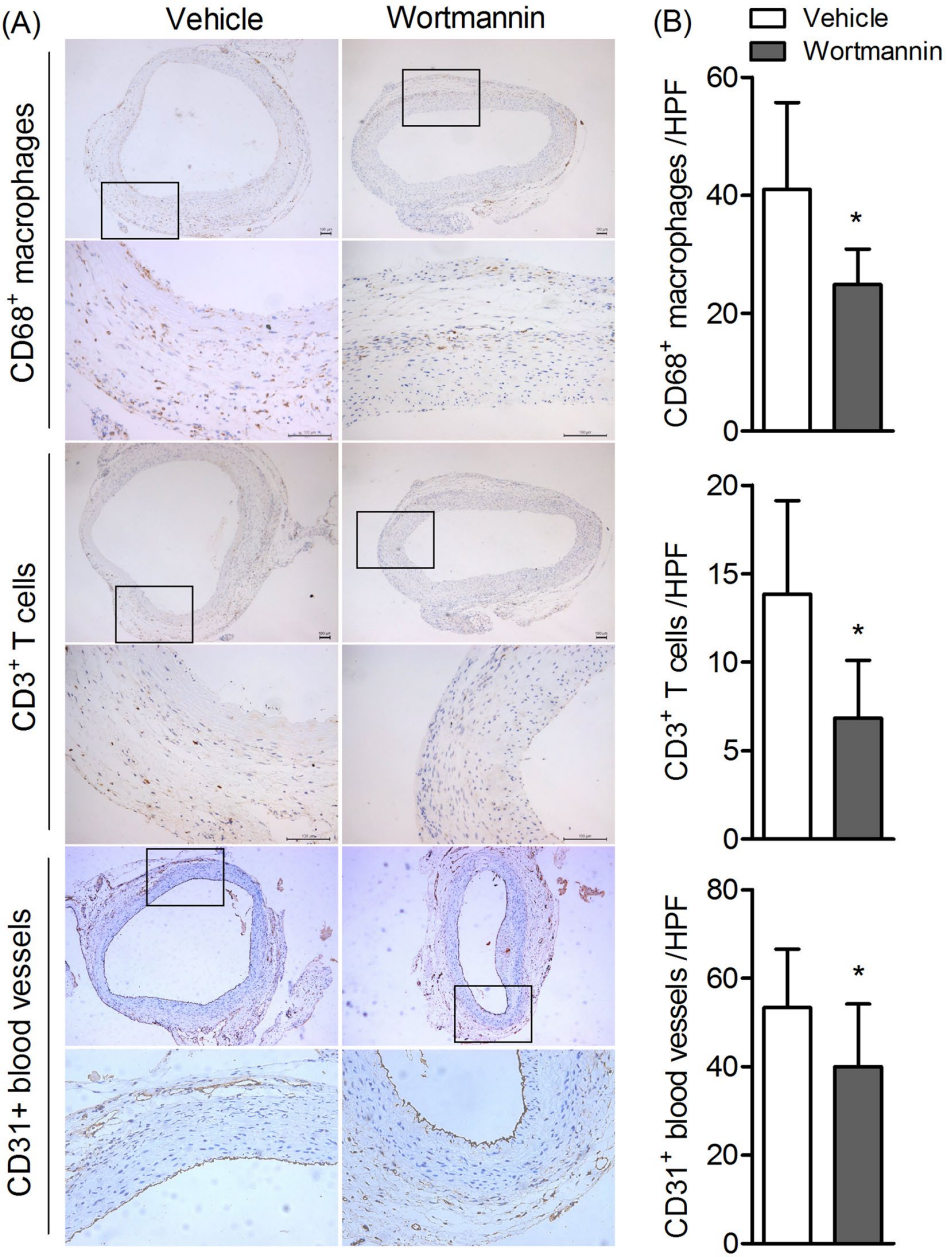
PI3K inhibitor treatment attenuates the inflammatory cell infiltration in AAAs. Aortic sections from rats 28 days after PPE infusion were stained with antibody against CD68, CD3 and CD31 respectively. (**A**) Representative aortic histology images for macrophages, T cells and MVD cells. Original magnification: ×50 and ×200. (**B**) CD68+ macrophages, CD3+ T cells, CD31+ blood vessels in media and adventitia were counted and data present as mean and SD per HPF (200×). n = 9–12 in each group. Nonparametric Mann-Whitney test. *P < 0.05 vs vehicle group. HPF, High-power field.

To further investigate the mechanism by which PI3K modify aneurysm pathogenesis, tissue immunostaining was used to evaluate the presence and magnitudes of inflammation cell infiltration. The histological analysis revealed that macrophages, T cells, blood vessels infiltration were significantly reduced in rats treated with Wortmannin (*P* < 0.05)(Fig.  3A,B). This result indicated that PI3K inhibitor inhibits AAA progression through preventing inflammation cell infiltration.

### PI3K inhibitor treatment inhibits the expression of pAKT and VEGF in AAAs

**Figure 4:**
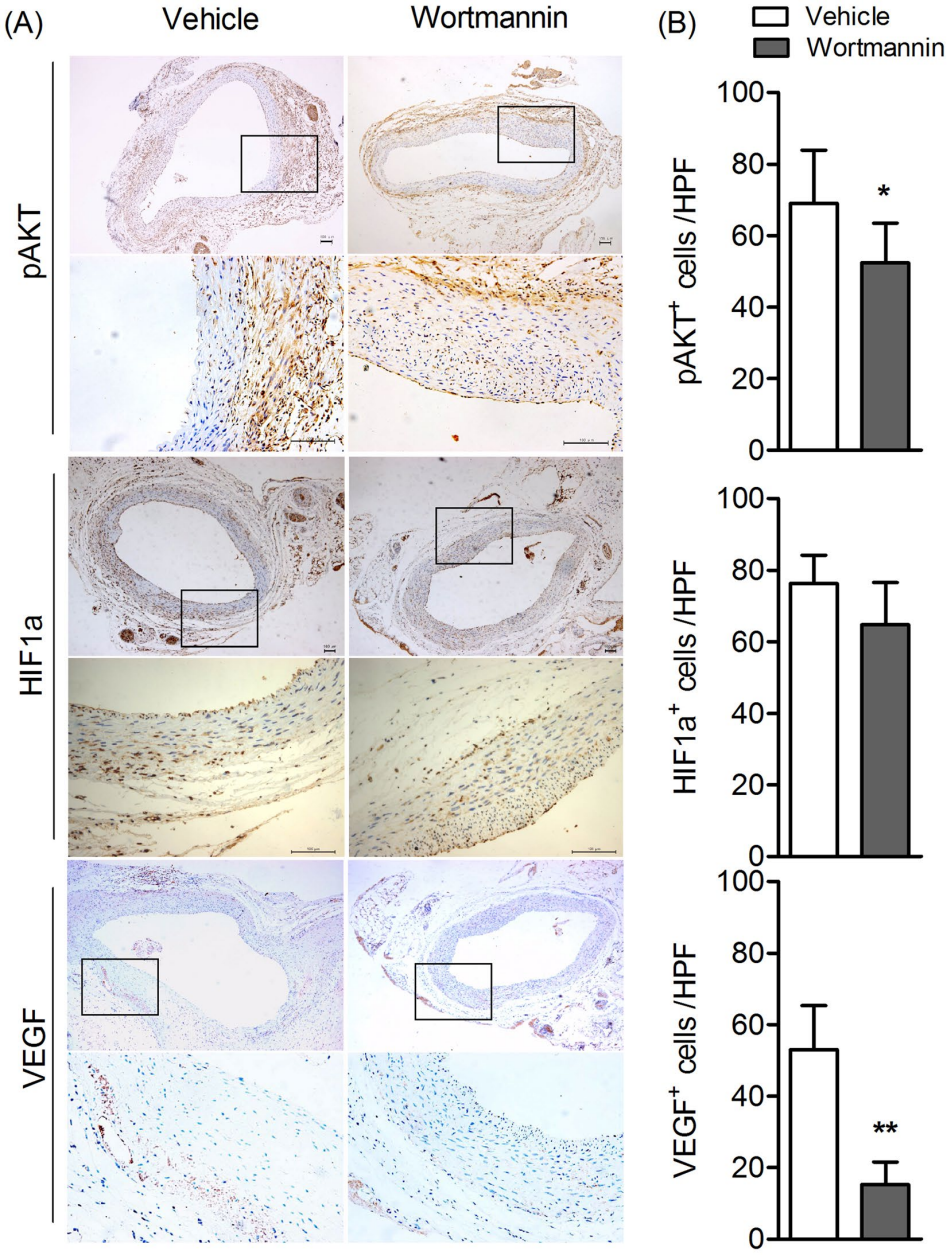
PI3K inhibitor treatment attenuates the expression of pAKT and HIF1a/VEGF in AAAs. Aortic sections from rats 28 days after PPE infusion were stained with antibody against CD68, CD3 and CD31 respectively. (**A**) Representative aortic histology images for pAKT, HIF1a and VEGF staining. Original magnification: ×50 and ×200. (**B**) pAKT^+^ cells, HIF1a^+^ cells, VEGF^+^ cells in media and adventitia were counted and data present as mean and SD per HPF (200×). n = 9–12 in each group. Nonparametric Mann-Whitney test. *P < 0.05 and **P < 0.01 vs vehicle group. HPF, High-power field.

Next, aneurysmal aorta tissue immunostaining was performed to investigate the mechanism of progression of AAA mediated by PI3K. Aorta tissues in PPE group or Vehicle group were stained with pAKT, HIF1a and VEGF or negative control antibody. As shown in Fig.  4A,B, Wortmannin inhibit the AKT phosphorylation during AAA progression (*P* < 0.05). Meanwhile, the general tendency of expression of HIF1a in Wortmannin treatment group was decreased as compared to Vehicle treatment group, although did not reach statistical significance (*P* > 0.05). Whereas the expression of VEGF was significantly decreased in aneurismal aorta tissue after Wortmannin treatment (*P* < 0.05). Together, these results indicated that PI3K inhibitor treatment inhibit AAA progression may be through inhibition of pAKT/VEGF signal pathway.

### pAKT expressed mainly in the infiltrating macrophage in the aneurismal aorta

**Figure 5:**
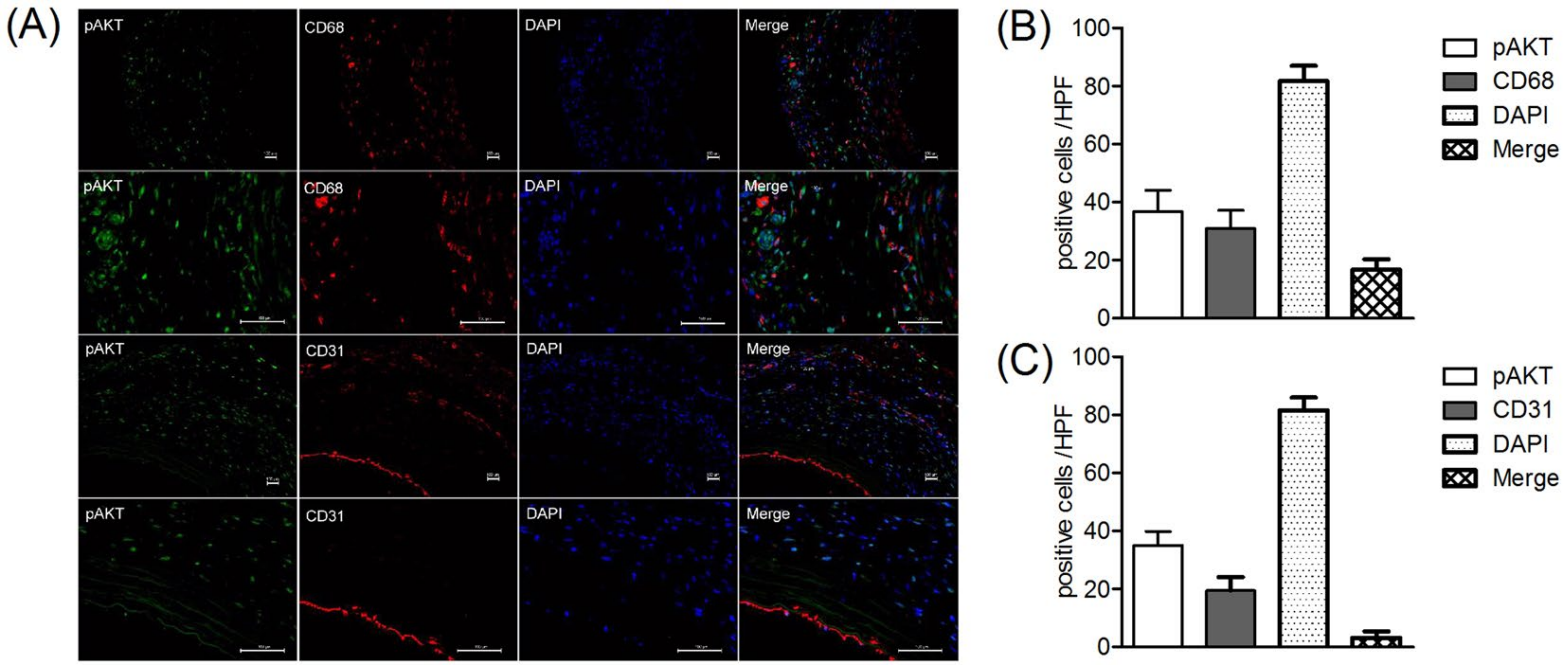
pAKT mainly expressed in macrophages in AAA tissues. Aortic sections from rats 28 days after PPE infusion were stained with antibody against pAKT and CD68 or CD31 respectively. (**A**) Antibody against pAKT was conjugated with Alexa Fluor 488 (green color) and antibody against CD68 or CD31 was conjugated with Alexa Fluor 546 (red color). Cell nucleus was stained with DAPI (blue color). Images obtained from fluorescence microscope were merged to locate the expression of pAKT in AAA tissues. (**B**) Positive cells of pAKT, or CD68, or DAPI or pAKT and CD68 cells in media and adventitia were counted and data present as mean and SD per HPF (200×). (C) Positive cells of pAKT, or CD31, or DAPI or pAKT and CD31 cells in media and adventitia were counted and data present as mean and SD per HPF (200×). HPF, High-power field..

To investigate the functional pattern of PI3K in aneurismal aortae, triple fluorescent staining was employed. Macrophage, or endothelial cells are stained with antibody conjugated with Alexa Fluor 546 (red color); pAKT protein are stained with antibody conjugated with Alexa Fluor 488 (green color); cell nucleus are stained with DAPI (blue color). As shown in Fig.  5, pAKT expressed mainly in the infiltrating macrophages in the aneurismal aorta. These results indicated the possibility of inhibiting the development of AAAs through inhibition of pAKT in macrophage by Wortmannin, however, this result need further confirmation.

### mRNA levels of PI3K increased in human AAA and correlated with diameter of human AAA

**Figure 6:**
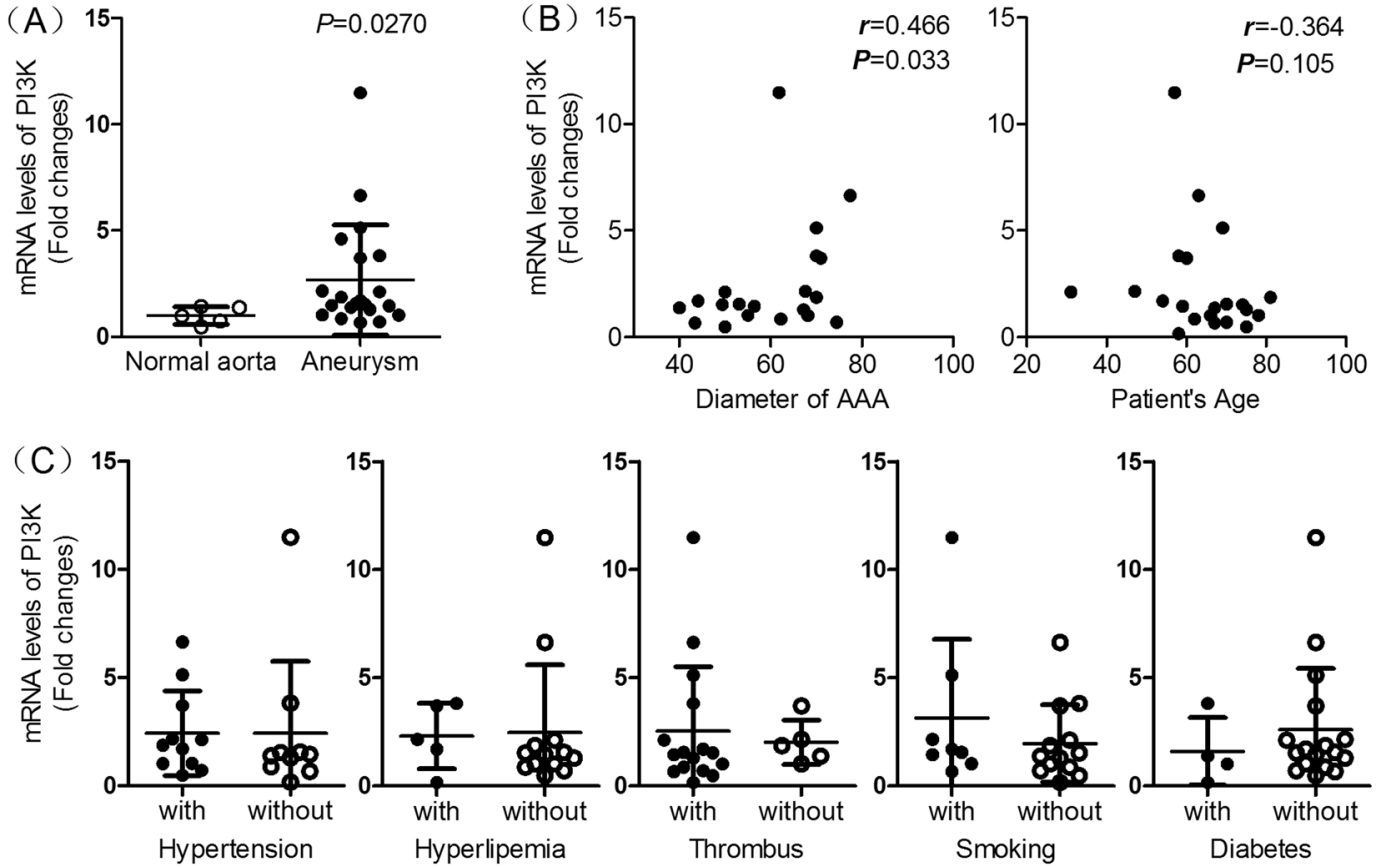
The correlation between PI3K expression and AAA clinical characteristics. (**A**) mRNA levels of PI3K in human aneurismal tissues and normal aorta tissues were determined by Realtime-PCR. Non-parametric Mann-Whitney test. (**B**) The correlation between mRNA levels of PI3K and diameter of AAA or age of AAA patients were determined. Spearman rank correlation test. (**C**) The correlation between PI3K expression and AAA clinical characteristics such as thrombus, diabetes, hypertension, hyper lipemia and smoking history were determined. Non-parametric Mann-Whitney test.

To explore the potential translational value of PI3K during the progression of human AAA, we examined the mRNA expression of PI3K in human AAA. Twenty-four cases of aneurysm tissues and six cases of normal aorta tissues were collected and Realtime-PCR was performed. As shown in Fig.  6A, the mRNA expression of PI3K was elevated in human aneurismal aorta as compare to normal aorta (*P* < 0.05). Furthermore, there is a positive correlation between mRNA levels of PI3K and diameter of AAA (*P* < 0.05) (Fig.  6B). Meanwhile, the expression of mRNA levels of PI3K showed a general tendency of positive correlation with medical history such as thrombus and smoking, whereas negative correlation with medical history such as diabetes (*P* > 0.05). Collectively, these results suggested a potential translational value of PI3K in inhibition of human AAA.

### Expression of pAKT protein increased in human AAA

**Figure 7:**
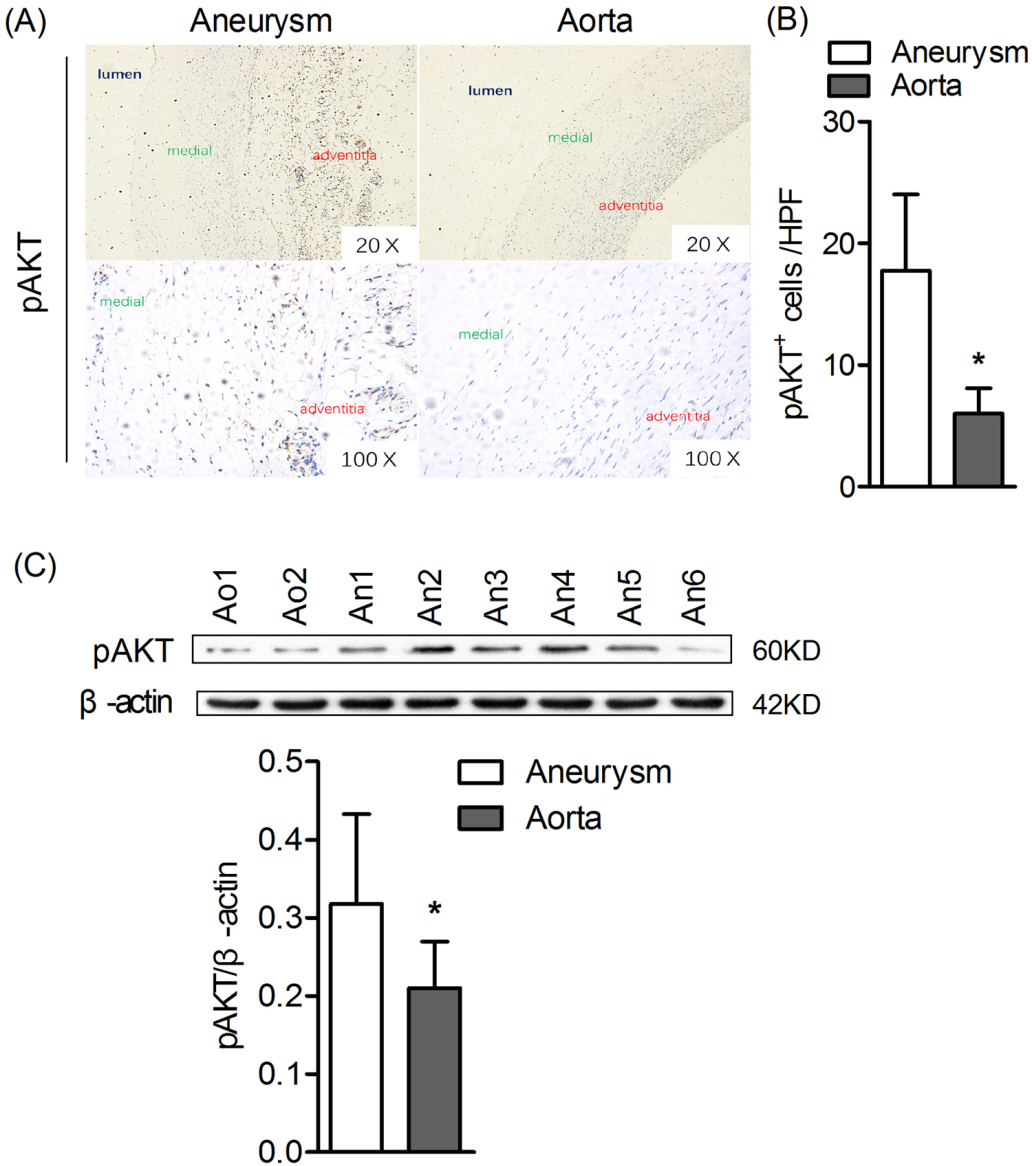
Increased expression of pAKT protein in human AAA. (**A**) Representative human aortic histology images for pAKT staining. Original magnification: ×20 and ×100. (**B**) pAKT^+^ cells in media and adventitia were counted on each HPF (100×), and data present as mean and SD per HPF. n = 24 for aneurysm group and n = 6 for normal aorta group. Nonparametric Mann-Whitney test. *P < 0.05 vs aneurysm group. (**C**) Protein levels of pAKT in human aneurismal tissues and normal aorta tissues were determined by Western Blot. Ao is representative for aorta and An is representative for aneurysm. Original uncropped western blots are provided in a Supplementary Figure  1. Data present as mean and SD, n = 24 for aneurysm group and n = 6 for normal aorta group. Nonparametric Mann-Whitney test. *P < 0.05 vs aneurysm group. HPF, High-power field.

To further explore the potential function of PI3K in aneurysm, the expression of phosphorylated AKT (pAKT) was analyzed in aneurysm tissues and normal aorta tissues. As shown in Fig.  7A,B, pAKT protein analyze using IHC revealed pAKT mainly expressed in adventitia and significantly increased in human AAA tissues compare to normal aorta. To verify this result, analysis of pAKT protein by Western blot was performed. Anticipatively, expression of pAKT elevated significantly in aneurysm tissues as compared with normal aorta tissues (*P* < 0.05)(Fig.  7C). Collectively, these results supported a potential translational value of PI3K in inhibition of human AAA

## Discussion

Accumulated documents indicated AAA is characterized by the following pathological features: intense local inflammatory infiltration and cytokine production, elevated expression of MMP, reduced SMCs, and ECM degradation^16,17^. Experimental AAA model created by transient intra-aortic infusion of PPE was extensively employed for the aneurysm research because this model correctly simulates the pathological features of human AAAs among all wildly used experimental AAA model respectively. Based on the classical rat experimental AAA model^14^, a shorter time (30 minutes) infusion of PPE was applied in our present rat experimental AAA model^15^, this modification of infusion time decreased the high mortality and morbidity of hind limb ischemia^13^. Here, all rats have developed AAA in day 28 after PPE infusion (9/9, 100%) indicated a successful establishment of rat experimental AAA model in our present study.

It is well known that the PI3K/AKT pathway is activated in most of human cancers, which regulate numerous cellular functions including proliferation, adhesion, migration, invasion, metabolism, and survival. Recently, documents suggested an important role of PI3K in inflammatory cells infiltration and angiogenesis^18^. Our present study revealed an up-regulated expression of PI3K and pAKT in abdominal aorta in rats received infusion of PPE as compare to rats received infusion of PBS, these results consist with previous finds that phosphorylation of AKT was significantly increased in male mice PPE AAA model^19^. In fact, our previous results showed an increased level of PI3K in serum of human abdominal aortic aneurysm (unpublished data). Collectively, those results suggested a potential relationship between PI3K function and initiation and progression of AAAs.

Lots of compounds targeting PI3K/AKT pathway has been used in clinical trial for human cancer treatment^8,20–22^. Wortmannin is a kind of currently known inhibitor that shows high specificity for PI3K, which binds to the p110 catalytic subunit of PI3K, noncompetitively and irreversibly inhibiting(IC_50_ = 5 nm) the enzyme^23^. Early findings confirm and extend prior observations regarding wortmannin in the treatment of cancer^24,25^. As recently document indicates a potential important role of PI3K/AKT pathway in inflammation^26,27^, it is rational to question whether PI3K inhibitor could limits the progression of AAA. Our results demonstrate for the first time that Wortmannin, a prototype and classical PI3K inhibitor, suppresses PI3K/AKT pathway and prevents the formation and progression of experimental abdominal aortic aneurysm in rats. PPE Rats administrated with Wortmannin showed an alleviative elastin and SMC destruction as compare to Vehicle. This result was in accordance with another research, in which administration with Wortmannin could prevent the progression of ascending aortic aneurysms in mice^28^.

Ghosh *et al*. demonstrated that mice administrated with LY294002, another classical AKT phosphorylation inhibitor, showed less disruption of elastin fibers in aorta after PPE infusion compared with those mice received Vehicle treatment^19^. Meanwhile, LY294002 relieved macrophage infiltration and decreased the level of MMP9 in aorta. However, treatment of LY294002 did not completely abolish AAA formation after elastase perfusion in mice. We speculated that the reason is either the low dose of LY294002 that was used or the timing of its delivery started 4 days after PPE infusion. Thus, in our present study, Wortmannin was employed to inhibit PI3K/AKT pathway. Also, rat was administrated with Wortmannin started at day 0 after PPE infusion. Our results, consistent with the above results, indicated PI3K as a potential therapeutic target for AAA.

Our results showed that PI3K inhibitor treatment attenuates the inflammatory cell infiltration in experimental AAAs. The Wortmannin can reduce influx of the CD3^+^ T cells and CD31^+^ blood vessels as previous described^26,29^. In our study, PI3K inhibition treatment significantly reduced aortic macrophage infiltration while suppressing AAA formation and progression, as the CD68 ^+^ macrophages in Wortmannin group is less than the Vehicle one. Furthermore, our results revealed that Wortmannin inhibit the phosphorylation of AKT, accompanied with decreased expression of VEGF in aneurismal aorta. Especially, expression of HIF1a showed a downtrend in Wortmannin group, although did not reach statistical significance. In consideration of rapid degradation of HIF1a protein under normal oxygen conditions, It can explain the reason why our IHC results did not reach statistical significance^30^. HIF1a is a principle regulator of hypoxic adaptation, regulating gene expression involved in glycolysis, erythropoiesis, angiogenesis, proliferation, and stem cell function under low O2. Interestingly, increasing evidence accumulated over recent years suggests an important regulatory role for HIF1a in inflammation^31^. Previous experiments demonstrate the ability of the HIF1a inhibitor to both suppress experimental AAA initiation and stabilize existing aneurysms, through mechanisms likely related to impair mural macrophage infiltration and angiogenesis^20,32^. Our present study revealed that Wortmannin inhibit the expression of pAKT and VEGF in aneurismal aorta, which suggest a potential mechanism of limiting AAA formation and progression through targeting PI3K/AKT pathway^33^. Certainly, to clarify the exact role of HIF1a in PI3K/AKT pathway during aneurysm progression, further research is anticipated.

To date, many pharmacologic approaches have been proven to be frustratingly ineffective in limiting AAA disease progression in clinical trials because of the wild difference between experimental AAA model and human AAA disease^4^. To evaluate the translational value of PI3K inhibitor, we detected the expression of PI3K in human aneurismal aorta. Furthermore, to verify the function of PI3K, pAKT levels in human aneurismal aorta tissues was also examined. Our encouraging results demonstrated an up-regulated mRNA expression of PI3K in human aneurismal aorta and a positive relationship between its expression and diameter of AAA. This result, along with the increased pAKT in aneurismal aorta, highlights a potential translational value of PI3K in inhibition of human AAA.

However, there are some limitations in our study. The stabilizing effect of PI3K inhibitor therapy on existing AAA has not been evaluated. Most prior AAA inhibition studies in experimental models begin therapy prior to aneurysm initiation, a situation at odds with the clinical reality of AAA diagnosis and pre-surgical disease management. Thus, to evaluate the stabilizing effect of PI3K inhibitor for already existing aneurysm is anticipated.

Our present study demonstrates that inhibition of PI3K/AKT pathway could prevent the formation and progression of experimental abdominal aortic aneurysm in rats. Wortmannin limits experimental AAA progression through preventing elastin degradation and inflammatory cells infiltration. These findings suggest that PI3K inhibitor may hold substantial translational value for AAA diseases.

## Materials and Methods

### Rats and Experimental AAAs creation

The experiment was carried out on male Sprague-Dawley rats, aged 8 to 12 weeks and weighed 250–300 g. All rats were housed under a 12-hour light/dark cycles with standard diet and water. AAAs was created by transient intra-aortic infusion of PPE for male S-D rats as previous described^15^. Briefly, after using chloral hydrate anesthesia by intraperitoneal injection, the infrarenal aorta was isolated from the level of the left renal vein to the iliac bifurcation via median laparotomy. Following exposure, intra-aortic infusion was performed for 30 minutes with type I porcine pancreatic elastase (1.5 units/ml freshly prepared in phosphate-buffered saline (PBS), catalog # E1250–100MG, Sigma-Aldrich, ST. Louis, MO) at a certain dose of 0.5 ml containing 50 U. Following infusion and removal of residual elastase, the tubing was withdrawn and aorta closed with 10–0 nylon suture. After laparotomy closure and surgical recovery, rats were housed in separate cages with free access to chow and water. The diameter of aorta was measured directly underwent laparotomy at day 0, day 7 and day 28 after PPE infusion. Vernier caliper was employed to measure dilation of the AAAs and an AAA was defined as a 50% increase in aortic diameter compared with baseline. All animal experiments were carried out in accordance with the animal care guidelines and regulation of Department of Laboratory Animals in Central South University and approved by the Department of Laboratory Animals in Central South University.

### Drug treatment

Wortmannin (Catalog No. S2758, Selleck, China), a classical PI3K inhibitor was employed to investigate whether inhibition of PI3K pathway could prevent the formation of aneurysm in the rat AAAs model. Those rats were divided into two groups as following: Vehicle group in which rats received elastase perfusion and treated with DMSO by intraperitoneal injection; Wortmannin group in which rats received elastase perfusion and treated with intraperitoneal injection of Wortmannin at a dose of 0.5 mg/kg/day (dissolved in DMSO) as previously reported from day 0 to day 28 after PPE infusion^28,34,35^.

### Elastin and Immunohistochemically Staining

Rats were sacrificed at day 28 after PPE infusion. Aorta were harvested and fixed in 10% neutral buffered formalin, tissues was embedded in paraffin and cut into sections (5 um thickness). Hematoxylin-Eosin, Masson and Elastic Van Gibson (EVG) staining were performed to evaluate elastin and SMC destruction^36,37^. Tissue immunostaining was performed using a standard biotin-streptavidin peroxidase procedure as previously described^38,39^. The primary antibodies for immunohistochemistry included as following: rabbit anti-rat PI3K polyclonal antibody (Cell Signaling Technology, Danvers, MA, USA), rabbit anti-rat pAKT Phospho (Ps473) polyclonal antibody (Goodbio Technology CO, LTD, Wuhan, China), rabbit anti-rat VEGF polyclonal antibody (Goodbio Technology CO, LTD, Wuhan, China), rabbit anti-rat HIF-1a polyclonal antibody (Goodbio Technology CO, LTD, Wuhan, China), rabbit anti-monoclonal CD3 polyclonal antibody (ZSGB-bio, Beijing, China), rabbit anti- monoclonal CD31 polyclonal antibody (ZSGB-bio, Beijing, China), mouse anti-monoclonal CD68 polyclonal antibody (ZSGB-bio, Beijing, China). Negative controls were carried out by omitting the primary antibody. Destruction of medial elastin and SMCs was graded as 0 to 5 as previously described^37^. Data on mural macrophage infiltration and angiogenesis are provided as the number of macrophages, T cells and CD31+ blood vessels per high-power field (HPF), respectively^40^. Staining located in the medial layer and adventitia is considered effective. Five HPF images per section were evaluated by two pathologists individually and average count were taken for each rat.

### Clinical abdominal aortic aneurysm tissue and normal aorta tissue specimens

The protocol of collection for clinical abdominal aortic aneurysm tissue and normal aorta tissue specimens was approved by the Ethics Committee of the Xiangya hospital, Central South University (Issued from 28 May, 2012). Following this protocol, informed consent was obtained from all participants and/or their legal guardians. The fresh samples of abdominal aortic aneurysm tissues were obtained from 24 patients with AAA who underwent open surgery at Xiangya Hospital of Central South University (CSU). The normal aorta tissues were obtained from six persons who were donor for liver transplantation at Xiangya Hospital of Central South University (CSU) and Zhongshan Hospital of Fudan University. The informed consent was obtained from all participants and/or their legal guardian/s. The specimens were immediately flash frozen in liquid nitrogen and stored at −80 °C for Realtime-PCR. The median age of the patients was 66 years, ranging from 31–81 year. The clinic parameter such as gender, aneurysm diameter, hypertension, hyper lipemia, diabetes, thromb us and smoking history were also collected, as shown in supplementary table 1. All methods involving human participants/donors were performed in accordance with the guidelines and regulation of Ethics Committee of the Xiangya hospital, Central South University.

### Real-Time Quantitative RT-PCR

Total RNA from human abdominal aortic aneurysm tissue was extracted using TRIzol reagent. cDNA was synthesized using RevertAid First Strand cDNA Synthesis Kit, followed by amplification using SYBR FastStart Universal. b actin expression was as internal reference. Gene expression levels were expressed as fold changes relative to normal aorta. PI3K: Forward:5′-TACACTGTCCTGTGCTGGCTACT-3′, Reverse: 5′-GAGATTCCCATGCCGTCGTA-3′; b-actin: Forward: 5′-CACCCAGCACAATGAAGATCAAGAT-3′, Reverse: 5′-CCAGTTTTTAAATCCTGAGTCAAGC-3′. The fold change in expression of each target mRNA relative to actin was calculated based on the threshold cycle as 2^−∆(∆Ct)^, where ∆Ct = Ct_target_ − Ct _actin_ and ∆(∆Ct) = ∆Ct_aneurysm_ −∆Ct_normal aorta_. All reagents were purchased from, or synthesized at, Goodbio Technology CO, LTD.

### Western Blot

Tissues were lysed in a lysis buffer. Extract equivalent to 100 µg of total protein was separated on 10% polyacrylamide gels and transferred to NC membrane. The membrane was incubated with primary antibody (Rabbit anti-Phospho-Akt (Ser473) mAb, #4060, Cell Signaling Technology, Danvers, MA, USA, diluted at 1:2000) or (Mouse anti-b-actin mAb, #60008-1-Ig, proteintech, Rosemont, IL, USA, diluted at 1:5000); followed by incubation with a 1:6000 dilution of horseradish peroxidase-linked second antibody (proteintech, Rosemont, IL, USA). Then the membrane was washed and treated with western blotting luminal reagent (Thermo, USA) to visualize the bands. The exposed film was scanned and the software was analyzed with quantity one professional grayscale analysis software.

### Statistical analyses

All continuous data was reported as the mean ± standard deviation (SD). The nonparametric Mann-Whitney test, or two-way ANOVA followed by Newman-Keuls post-test, were used to determine differences between groups according to data characteristics. Difference in aneurysm incidence was determined by Kaplan-Meier analysis. All statistical analyses were performed using the Prism Version 5.0, GraphPad Prism Software, Inc, San Diego, CA. P < 0.05 was considered statistically significant.

### Data Availability

The datasets generated during and/or analyses during the current study are available from the corresponding author on reasonable request.

### Statement of human tissues collection

The protocol of collection for clinical abdominal aortic aneurysm tissue was approved by the Ethics Committee of the Xiangya hospital, Central South University (Issued from 28 May, 2012). Following this protocol, informed consent was obtained from all participants and/or their legal guardians. The normal aorta tissues were obtained from persons who were brain dead organ donor for transplantation at Xiangya Hospital of Central South University (CSU) and Zhongshan Hospital of Fudan University. The protocol of collection was approved by the Ethics Committee of the Xiangya hospital, Central South University and the Ethics Committee of Zhongshan Hospital of Fudan University. All donor information is registered on the unified organ donation website and the informed consent was obtained from all participants and/or their legal guardian/s. NO tissues were procured from prisoners.

## Electronic supplementary material

Dataset 1

Original uncropped western blots of the images reported in Fig.7C
