# Supplementary material for: Inhibition of Phosphatidylinositol 3-kinease suppresses formation and progression of experimental abdominal aortic aneurysms
Source: Sci Rep. 2017 Nov 9;7:15208. doi: 10.1038/s41598-017-15207-w (PMC5680315; doi:10.1038/s41598-017-15207-w)
Supplement: Supplementary file 2 — Original uncropped western blots of the images reported in Fig.7C [file 41598_2017_15207_MOESM2_ESM.pdf]

Inhibition of Phosphatidylinositol 3-kinease suppresses formation and progression of experimental abdominal aortic aneurysms

Jing Yu, Rui Liu, Jianhua Huang, lixin Wang, Wei Wang*

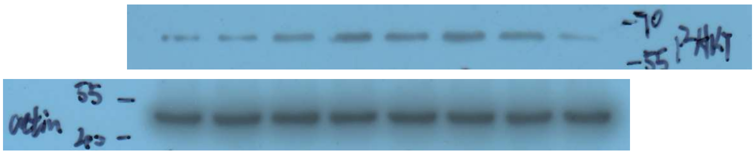

Supplementary Figure 1: Original uncropped western blots of the images reported in Fig.7C
